# Phenylbenzohydrazides Obtained from Isatoic Anhydride Present Anti-Inflammatory Activity In Vivo and In Vitro

**DOI:** 10.3390/biom12121901

**Published:** 2022-12-19

**Authors:** João Pedro Barros Paiva, Millena Santos Cordeiro, Patricia Ribeiro Carvalho França, Luiz Octavio Pereira Branco, Isabela Souza Santos, Nanashara Figueiredo Reis, Patrick Pedro Pimentel, Thais Biondino Sardella Giorno, Evanoel Crizanto Lima, Patricia Dias Fernandes

**Affiliations:** 1 Laboratório de Farmacologia da Dor e da Inflamação, Programa de Pesquisa em Descoberta de Fármacos, Instituto de Ciências Biomédicas, Centro de Ciências da Saúde, Universidade Federal do Rio de Janeiro, Rio de Janeiro 21941-901, Brazil; 2 Laboratório de Catálise e Síntese de Substâncias Bioativas, CM UFRJ-Macaé, Instituto Multidisciplinar de Química, Universidade Federal do Rio de Janeiro, 27971-525 Macaé, Brazil

**Keywords:** phenylbenzohydrazides, isatoic anhydride, inflammation, anti-inflammatory substance, carrageenan-induced inflammation, nitric oxide

## Abstract

Background: Despite the existence of a wide variety of anti-inflammatory drugs, the vast majority are classified as steroidal or non-steroidal. Both classes present a variety of side effects that limit usage. Thus, the search for new molecules with anti-inflammatory potential is still important. Methods: Five phenylbenzohydrazides were synthetized and evaluated in pre-clinical models of acute inflammation in vivo and in vitro. Results: The new substances (INL-06, -07, -10, and -11), as well as AISCT, significantly reduced cell migration induced by carrageenan. It was also observed that all INLs inhibited protein extravasation as well as cytokines (IL-6, IL-1β, and TNF-α) and nitric oxide (NO) production. The INL-11 was demonstrated to be the most potent, since the inhibition observed in several parameters was significant even when compared with dexamethasone. In vitro INLs also reduced cytokines and NO production and inducible nitric oxide (iNOS) enzyme activity. The INL-11 was the most effective in reducing cell migration in vitro. Conclusions: Our data suggest that these substances are suitable for further development into a new series of compounds that could lead to new hits and future drug prototypes for anti-inflammatory conditions.

## 1. Introduction

The complexity of the regulation of inflammation and the overlapping between different mediator systems presents challenges to the development of highly effective, tolerable, and safe therapeutics. Inflammatory diseases are often controlled only partly, even by cocktails of substances [1]. 

Neutrophils and macrophages the one of the most important cells in defending the organism from an infectious disease. These cells release inflammatory mediators in an attempt to protect against and resolve of the inflammatory process. Mediators, such as cytokines, nitric oxide (NO), chemokines, vasoactive amines, leukotrienes, and prostaglandins (PGs) are liberated by cells; however, they can promote fever, redness, edema, and pain [1]. Cytokines and chemokines produced by resident cells recruit, through their receptors, circulating neutrophils [1]. These neutrophils transmigrate through the endothelium, reach the inflamed tissue, where they degranulate, generate reactive oxygen species (ROS), and produce other mediators, such as NO and PGs, amplifying the inflammatory response [1].

The most effective functional group of drugs to treat inflammation is glucocorticoids (SAID). These drugs have the potential to impact on the expression of at least 6500 genes [2]; their non-steroidal counterpart, non-steroid anti-inflammatory drugs (NSAID) have a narrower, but nonetheless broad impact, in part due to the range of cyclo-oxygenase products that are affected. However, both the efficacy and the adverse effect profile of glucocorticoids are related to their breadth of activity. Thus, the search for new drugs that could act in a different system from NSAID and SAID continues to be an objective for pharmacologists and medicinal chemists.

The phenylbenzohydrazides reported in this paper were designed through molecular simplification of the arylidenehydrazinyl-quinazolinones, which showed anti-inflammatory activity [3]. To the best of our knowledge, the anti-inflammatory activity of benzohydrazide has not been studied. Different substituents were introduced to phenyl hydrazide moiety, such as electron-withdrawing chlorine, bromine, and fluorine groups, as well as an electron-donating methyl group, aiming to investigate their electronic influence on anti-inflammatory activity. Isatoic anhydride have also been tested for being the starting material and presenting structural similarity with molecules previously studied by our group [4,5].

## 2. Materials and Methods

### 2.1. Synthesis of INLs

*Synthesis of INL-06* was as follows: A mixture of isatoic anhydride (1 g, 6.13 mmol) and phenyl hydrazine (0.663 g, 6.13 mmol) in ethanol (60 mL) was heated under reflux for 2 h. Reaction was accomplished by thin layer chromatography (TLC). The resulting solid was filtered and air-dried to furnish the product in an 80% yield. Its structure was confirmed by proton nuclear magnetic resonance (^1^H NMR), gas chromatography coupled to mass spectrometry (GC-MS), and melting point according to the literature. Here, mp = 170–171 °C (as reported, Shemchuk, 2008; ^1^H NMR (DMSO-d6, 400 MHz) δ ppm 10.10 (s, 1H, NH), 7.79 (s, 1H, NH), 7.66 (d, 1H, *J* = 7.8, ArH), 7.22–7.13 (m, 3H, ArH), 6.78 (d, 2H, *J* = 7.7, ArH), 6.75–6.69 (m, 2H, ArH), 6.56 (t, 1H, *J* = 7.8, ArH), 6.39 (s, 2H, NH_2_). The MS (EI) values were *m/z* 227 (24%), *m/z* 120 (100%), *m/z* 92 (21%), *m/z* 121 (8%), and *m/z* 77 (4%).

*Synthesis of INL-07, 09, 10 and 11* was as follows: NaOH (0.245 g, 6.13 mmol) was added to a solution of corresponding phenyl hydrazine (6.13 mmol) in ethanol (60 mL). The mixture was stirred for 5 min, and then isatoic anhydride (1 g, 6.13 mmol) was added. The reaction media was heated under reflux for 2 h. After this period, the resulting solid was filtered and air-dried to furnish the respective phenylbenzohydrazide. Their structures were confirmed by ^1^H NMR, GC-MS, and melting point according to the literature [6,7,8]. 

*INL-07*. Yield, 61%. White solid, mp = 198–199 °C (Anand, 2015); ^1^H NMR (DMSO-d6, 400 MHz) δ ppm 10.13 (s, 1H), 8.00 (s, 1H), 7.66 (d, 1H, *J* = 7.9Hz), 7.31–7.27 (m, 2H), 6.73 (d, 3H, *J* = 8.9 Hz), 6.54 (t, 1H, *J* = 8.0 Hz), and 6.40 (s, 2H). The MS (EI) values were as follows: *m/z* 305 (9%), *m/z* 307 (9%), *m/z* 120 (100%), *m/z* 92 (17%), and *m/z* 121 (12%).

*INL-09*. Yield, 60%. White solid; ^1^H NMR (DMSO-d6, 400 MHz) δ ppm 10.13 (s, 1H), 7.98 (s, 1H), 7.21–7.16 (m, 3H), 6.78 (d, 2H, *J* = 8.9Hz), (d, 1H, *J* = 9.2 Hz), 6.54 (t, 1H, *J* = 8.0 Hz), and 6.39 (s, 2H).The MS (EI) values were as follows: *m/z* 261 (15%), *m/z* 263 (5%), *m/z* 120 (100%), *m/z* 92 (18%), and *m/z* 121 (9%).

*INL-10*. Yield, 45%. White solid, mp = 140–142 °C (Yang, 2015); ^1^H NMR (DMSO-d6, 400 MHz) δ ppm 10.10 (s, 1H), 7.76 (s, 1H), 7.65 (d, 1H, *J* = 7.8 Hz), 7.18 (t, 1H, *J* = 8.4 Hz), 6.99 (t, 2H, *J* = 8.8 Hz), 6.80–6.77 (m, 2H), 6.72 (d, 1H, *J* = 8.2 Hz), 6.54 (t, 1H, *J* = 8.0 Hz), and 6.37 (s, 2H). The MS (EI) values were as follows: *m/z* 245 (24%), *m/z* 246 (4%), *m/z* 120 (100%), *m/z* 92 (21%), and *m/z* 121 (8%).

*INL-11*. Yield, 78%. White solid; ^1^H NMR (DMSO-d6, 400 MHz) δ ppm 10.15 (s, 1H), 7.69 (d, 1H, *J* = 7.9 Hz), 7.18 (t, 1H, *J* = 8.4 Hz), 7.14 (s, 1H), 7.03–6.99 (m, 2H), 6.72 (t, 2H, *J* = 8.6 Hz), 6.66 (t, 1H, *J* = 7.3 Hz), 6.55 (t, 1H, *J* = 8.0 Hz), 6.39 (s, 2H), and 2.21 (s, 3H). The MS (EI) values were as follows: *m/z* 241 (29%), *m/z* 242 (5%), *m/z* 120 (100%), *m/z* 92 (17%), and *m/z* 121 (9%).

### 2.2. Animals

Swiss Webster mice (25–30 g) were kindly donated by the Instituto Vital Brazil (Niterói, Rio de Janeiro, Brazil). Mice were maintained in a room with a light–dark cycle of 12 h, 22 ± 2 °C to 60% to 80% humidity, and with food and water provided ad libitum. Animals were acclimatized to the laboratory conditions for at least 1 h before each test on set and were used only once throughout the experiments. All protocols were conducted in accordance with the Guidelines on Ethical Standards for Investigation of Experimental Pain in Animals [9], and followed the principles and guidelines adopted by the National Council for the Control of Animal Experimentation (CONCEA), approved by the Ethical Committee for Animal Research (# 31/19 and 34/19). All experimental protocols were performed during the light phase. Animal numbers per group was kept at a minimum and at the end of each experiment mice were killed by a ketamine/xylazine overdose.

### 2.3. Drugs, Reagents, and Treatments

Dexamethasone, L-NMMA (L-NG-monomethyl arginine), Ara-C (cytosine arabinoside), MTT (3-(4,5-dimethyl-ltiazol-2-yl)-2,5-diphenyltetrazole), isatoic anhydride (AISTC), phenyl hydrazines, NaOH, and lipopolysaccharide were purchased from Sigma-Aldrich (St. Louis, MO, USA). Ethanol was purchased from Merck Inc. (São Paulo, Brazil). Cytokine kits were purchased from BD Biosciences (Franklin Lakes, NJ, USA), while a protein kit (Kit Pierce BCA™ Protein Assay) was purchased from Thermo Fisher Scientific, Inc. (Waltham, MA, USA).

The INLs were dissolved in dimethylsulphoxide (DMSO) to prepare 100 mg/mL stock solutions. For use, solutions were prepared from each stock solution using tween as the vehicle. Doses of 1 to 30 mg/kg (final volume of 0.1 mL per animal) were administered by gavage (without mixture with food), and final tween percentage did not exceed 1%. Dexamethasone (7.5 µmol/kg), L-NMMA (100 µM) were used as references drugs. The dose of dexamethasone and L-NMMA was chosen based on previous results obtained by our group when ED_50_ or IC_50_ (the dose or concentration that caused a 50% reduction in the effect in each procedure) were calculated. The control group was given vehicle (tween). All drugs were diluted just before their use.

### 2.4. Cell Culture

RAW 264.7 (ATCC # TIB-71) was grown in RPMI medium supplemented with 10% fetal bovine serum (from now on named as RPMI) and kept in a 5% CO_2_ incubator at 37 °C. An exchange of RPMI was carried out until cells reached 90% confluence and exponential growth. On the day of assays, cells were collected by scraping bottles and left to adhere in 96- or 12-well culture plates (2 × 10^6^ cells/mL). 

### 2.5. In Vitro Toxicity Test (Cell Viability)

In 96-well plates, RAW 264.7 cells were put to adhere at 37 °C, 5% CO_2_. After 30 min incubation with INLs (0.1, 1, or 10 µM), LPS (1 µg/mL) was added to some groups. After 24 h incubation (at 37 °C, 5% CO_2_), the supernatant was changed and MTT solution (5 mg/mL, 100 µL/well) was added. After 4 h incubation (at 37 °C, 5% CO_2_) supernatants were discarded and DMSO (100 µL/well) was added to solubilize the MTT formazan crystals which formed [10]. Absorbance was measured at a wavelength of 570 nm using a Flexstation reader (Molecular Devices, San Francisco, CA, USA). Control groups were composed of cells which received only RMPI plus DMSO.

### 2.6. In Vivo Toxicity Test

Different groups of animals received an oral administration of 100 mg/kg of INLs. After 24 h, mice were euthanized with ketamine (50 mg/kg)/xylazine (20 mg/kg). A sample of blood was collected in a heparinized tube. The femur was removed, the ends were cut, and the bone marrow from each femur was washed with 1 mL of saline (NaCl 0.9%) with heparin and collected. Samples of blood and bone marrow were submitted to a complete blood hemogram and cell count, respectively, in an automatic cell counter (PocH-100iV Diff, Sysmex, Kobe, Japan).

### 2.7. Carrageenan-Induced Inflammation into the Subcutaneous Air Pouch (SAP)

The protocol was based on the work Romano et al. [11] with modifications [12]. A subcutaneous air pouch was induced in each mouse’s back through an injection of 10 mL of sterile air. After 3 days, a new injection of 7 mL of sterile air was performed on the animals’ backs. On the sixth day, the animals were orally treated with vehicle, INLs (at different doses), or dexamethasone (7.5 µmol/kg) and, after 60 min, mice received an injection of saline (NaCl 0.9%) or carrageenan (0.5%, 1 mL) into the SAP. After 24 h the animals were euthanized, and the SAP was washed with 1 mL of saline. The exudate was collected for leukocyte counting and centrifuged at 1500 r.p.m., for 10 min, 4 °C. The supernatant was collected and stored at −20 °C for several dosages (see below).

### 2.8. Quantification of Proteins and Cytokines

To perform the quantification of proteins in the exudate obtained in the BAS experiment, a proper protein dosing kit, the BCA Protein Assay Reagent (Thermo Fisher Scientific, Inc., USA) was used. According to the manufacture protocols, 5 µL of the sample was incubated with 195 µL of the solution of reagents A and B in an oven at 37 °C for 30 min. The absorbance was measured at 570 nm, and the protein concentration was calculated using a standard curve made from BSA; the results expressed in µg/mL.

Quantification of cytokines was performed in the exudate collected from BAS and in the supernatant of RAW 264.7 cells, through an immunoenzymatic assay method (ELISA) using specific ELISA kits (BD OptEIA^TM^ Set mouse, B&D, USA). For the assay, 96-well plates were incubated with a specific capture antibody at 4 °C for 18 h. The plates were then washed (3 times), filled with blocking buffer, and incubated at room temperature for 1 h. Then, they were washed again (3 times) and 25 µL of the specific standard or samples were added. The plates were incubated for 18 h in an oven at 37 °C, after which they were washed (5 times) and incubated with the detection antibody plus the enzyme, with subsequent incubation for 1 h, at room temperature. Substrate solution was then added and incubated for 30 min and protected from light, followed by the addition of a stop solution (H_3_PO_4_ 1N, 12.5 µL/well). The absorbance reading was performed at 450 nm, and the concentration of cytokines was performed using a standard curve for each respective cytokine. Values were expressed as pg/mL.

### 2.9. Quantification of Nitric Oxide (NO) Production

Here, NO can interact with hemoglobin, decaying to nitrate (NO_3_^−^), and when its production occurs in vitro, it interacts with oxygen, decaying to nitrite (NO_2_^−^). The protocol for converting nitrate to nitrite was described by Bartholomew [13], with adaptations made by Raymundo et al. [12], and the technique used to measure NO_2_ was described by Green et al. [14]. The absorbance reading was carried out in a microplate reader (FlexStation, Molecular Devices, USA), at 540 nm. The sodium nitrite concentrations were calculated using a standard sodium nitrite curve.

### 2.10. Cell Migration In Vitro

To assess the effect of INLs on cell migration in vitro, RAW 264.6 cells were plated at 1 × 10^6^/well in 12-well plates (in a final volume of 2 mL) and, after 3 h, a scratch was made in the well with the aid of a tip. The wells were washed with RPMI, and 10^−5^ M of arabinoside (AraC; Sigma-Aldrich, Saint Louis, MO, USA) was added to prevent cell proliferation. The cells were treated with INLs (0.1, 1 or 10 µM). Photos were obtained immediately after treatment (0 h) and after 24 h, using an EvosM500 microscope (ThermoFisher). The area was measured with the aid of the ImageJ software. To obtain the results, three independent experiments were carried out.

### 2.11. Statistical Analysis

Each in vivo group was composed of 6 to 8 animals selected at random. In vitro experiments were repeated at least 3 times on different days, and each experimental group was studied in triplicate. The results were expressed as mean ± standard deviation, and statistical significance was calculated by analysis of variance (ANOVA) followed by Tukey’s post-hoc test, using the GraphPad Prisma 8.2 program. Here, *p* values less than 0.05 were considered significant.

## 3. Results

### 3.1. Chemistry

**Scheme 1:**
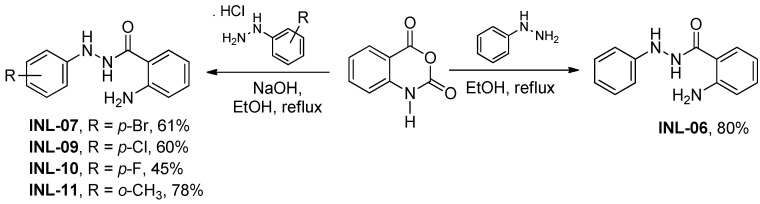
Synthesis of phenylbenzohydrazides.

First, the optimal conditions to obtain phenylbenzohydrazides (INL-06, 07, 09, 10, and 11) were investigated, due to the existence of several procedures described for obtaining INL-06 [6,7,8,15]. With the optimized condition on hand, isatoic anhydride was reacted with different phenyl hydrazines under reflux in ethanol for two hours to afford respective phenylbenzohydrazides (Scheme 1). We considered using sonication to improve our results, given its known use in heterogeneous systems [16,17,18]; however, all attempts to reduce the reaction time and improve the yields using ultrasound irradiation have been unsuccessful.

### 3.2. None of INLs Did Induce Any Toxicity after Oral Administration

**Figure 1:**
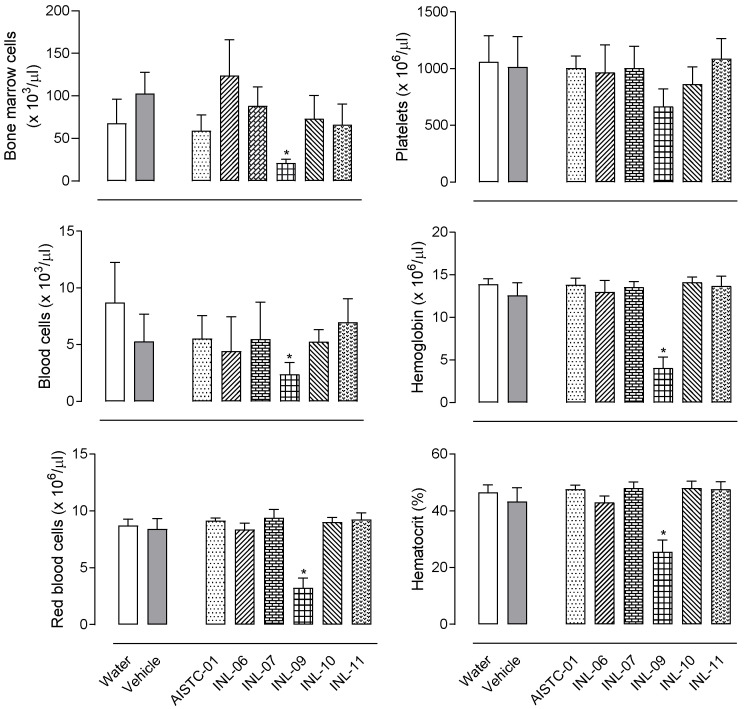
AISTC-01 and INL-06, -07, -10, and -11 did not present in vivo toxic effects. Substances were orally administered (at 100 µmol/kg) to mice. After 7 days, mice were sacrificed, and blood, and bone marrow were collected for measurements. Data are expressed as media ± standard error (*n =* 7). Statistical analyses were performed using ANOVA, following Newman–Keuls as a post-test. Here, * *p* < 0.01 when comparing the INL-09-treated group with the vehicle-treated group.

The oral administration of each INL to mice (at 100 µmol/kg dose) did not affect haematological parameters after 7 days. It can be observed in Figure 1 that AISTC-01, INL-06, INL-07, INL-10, or INL-11 did not affect the total number of cells in blood or bone marrow. No changes in the number of red blood cells, platelets, haemoglobin, and hematocrit were observed either, indicating that all these substances do not present any toxic effect at the dose tested. However, INL-09 administration resulted in a significant reduction in the number of red blood cells, platelets, haemoglobin, and hematocrit, suggesting a possible toxic effect. In view of these data, INL-09 was not further tested in the subsequent models.

### 3.3. Leukocytes Migration and Protein Extravasation Were Inhibited by INLs

**Figure 2:**
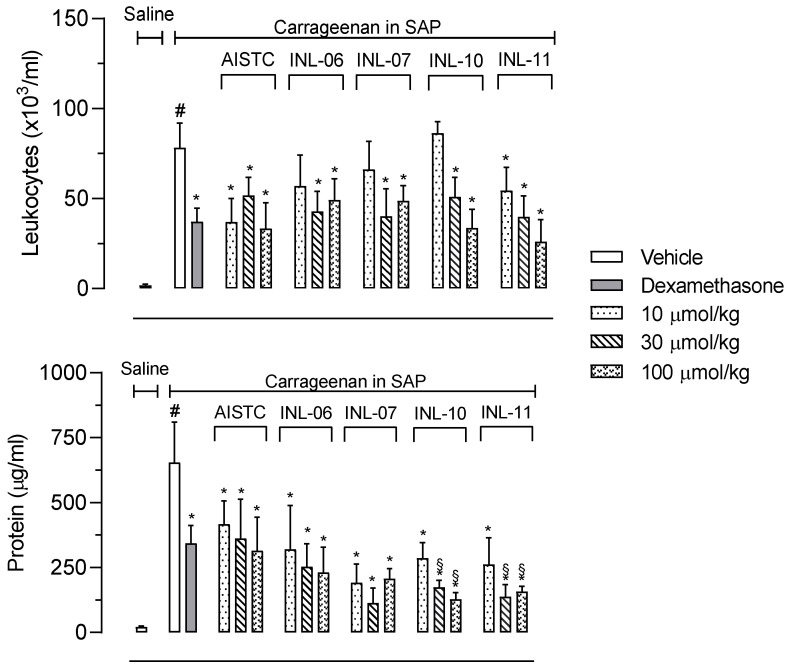
AISTC and INLs did not reduce leukocyte migration and the protein extravasated. Mice were pretreated with vehicle, AISTC, INL-06, INL-07, INL-10, or INL-11 (10, 30, and 100 µmol/kg, oral), or dexamethasone (7.5 µmol/kg, i.p.) 1 h before carrageenan (0.5%, *w/v*) injection into the subcutaneous air pouch. Exudates were collected 24 h after carrageenan injection. Results are expressed as media ± standard error (*n =* 5–7). Statistical analyses carried out using ANOVA following Newman–Keuls as a post-test. Here, # *p* < 0.01 when comparing the vehicle-treated group that received carrageenan in SAP with the vehicle-treated group that received saline in SAP, and * *p* < 0.01 when comparing the dexamethasone-, AISTC-, or INLs-pretreated groups that received carrageenan in SAP with the vehicle-treated group that received carrageenan in SAP. Furthermore, ^§^ *p* < 0.005 when comparing AISTC- or INLs-pretreated groups that received carrageenan in SAP with the dexamethasone-treated group that received carrageenan in SAP.

The first step was to evaluate if INLs could present an effect against cell migration in vivo. In this regard, mice were pretreated with different doses of each INL and, 1 h later, carrageenan was injected in the subcutaneous air pouch (SAP). The pretreatment with dexamethasone (a steroidal anti-inflammatory drug) inhibited almost 50% the number of leukocytes that migrated to the SAP. Furthermore, AISTC, as well as INL-11, significantly reduced cell migration at all doses used (10, 30 and 100 µmol/kg), while INL-06, INL-07, and INL-10 did present effects at 30 and 100 µmol/kg. It is interesting to note that effects observed were comparable to those with the positive control group, dexamethasone, even when the drug was intraperitoneally administered. In the same assay, we decided to quantify the protein extravasated to the exudate as being indicative of an increase in vascular permeability. Here, AISTC and INLs also reduced the protein accumulated in exudate, with all doses used. It is important to highlight that INL-10 and INL-11 significantly reduced protein extravasated even when comparing with the dexamethasone-treated group (Figure 2).

### 3.4. INLs Inhibited Inflammatory Mediators Produced in the Subcutaneous Air Pouch

**Figure 3:**
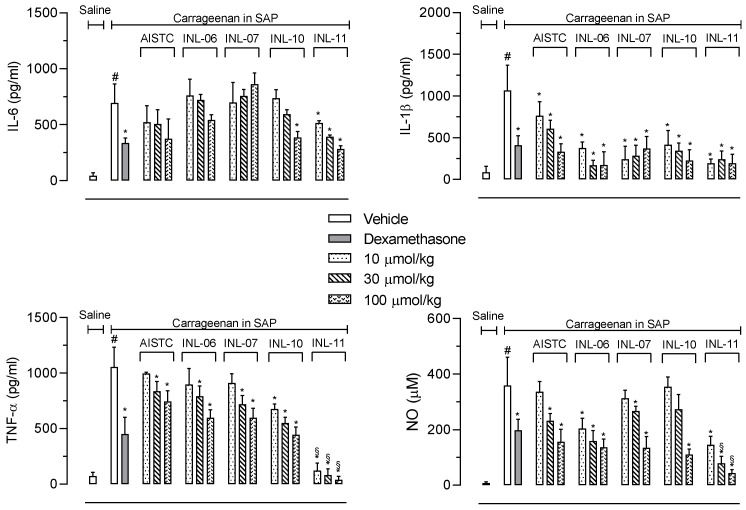
AISTC and INLs reduced inflammatory mediators’ production. Mice were pretreated with vehicle, AISTC, INL-06, INL-07, INL-10, or INL-11 (10, 30, and 100 µmol/kg, oral) or dexamethasone (7.5 µmol/kg, i.p.) 1 h before carrageenan (0.5%, *w/v*) injection into the subcutaneous air pouch. Exudates were collected 24 h after carrageenan injection. Results are expressed as media ± standard error (*n =* 5–7). Statistical analyses were carried out using ANOVA following Newman–Keuls as the post-test. Here, # *p* < 0.001 when comparing the vehicle-treated group that received carrageenan in SAP with the vehicle-treated group that received saline in SAP, and * *p* < 0.05 when comparing the dexamethasone-, AISTC-, or INLs-pretreated groups that received carrageenan in SAP with the vehicle-treated group that received carrageenan in SAP. Here, ^§^ *p* < 0.001 when comparing AISTC- or INLs-pretreated groups that received carrageenan in SAP with the dexamethasone-treated group that received carrageenan in SAP.

Next, we decided to quantify the production of cytokines and nitric oxide (NO) extravasated after carrageenan injection into the SAP. Figure 3 shows that IL-6 production was reduced only after pretreatment of mice with the higher dose of INL-10, whereas INL-11 presented a significant effect with all doses tested. In the other hand, neither AISTC nor INL-6 and INL-7 affected the production of this cytokine. 

When IL-1β was quantified, it could be observed that AISTC, as well as all INLs, significantly inhibited the cytokine production. The effects obtained after pretreatment of mice with INL-06, INL-07, INL-10, or INL-11 were comparable to those observed with dexamethasone. 

The most significant effect was in regard to the TNF-α production. Although all substances significantly reduced the cytokine levels, AISTC, INL-06, and INL-07 only presented a mild and significant effect with the two higher doses (30 and 100 µmol/kg). The most important effect was obtained with INL-11. This substance almost completely abolished the TNF-α production, even when the lowest dose (10 µmol/kg) was used. In these groups the TNF-α production was similar to that quantified in the negative control group (mice that received saline in the SAP).

We also measured nitric oxide (NO) accumulated in the exudate of the SAP. It is interesting to note a similarity to results obtained with TNF-α measurements. Although, AISTC, INL-06, INL-07, and INL-10 did reduce NO production, the effects of INL-11 were more pronounced. The INL-11 almost completely abolished NO production (Figure 3).

### 3.5. AISTC and INLs Did Not Present Cytotoxicity and Reduced Inflammatory Mediators’ Production and Cell Migration In Vitro

As INLs presented significant effects when administered orally to mice, we decided to further investigate if these substances could present effects when incubated in vitro. First of all, INLs were incubated with the RAW 264.A1 cell line (at 1, 10, or 30 µM) with or without lipopolysaccharide (LPS, 1 µg/mL). After 24 h the cytotoxicity was evaluated by MTT assay. Our data demonstrated neither AISCT nor INLs presented any alteration in cell viability, and is suggestive that they do not induce any cytotoxicity (data not shown). 

**Figure 4:**
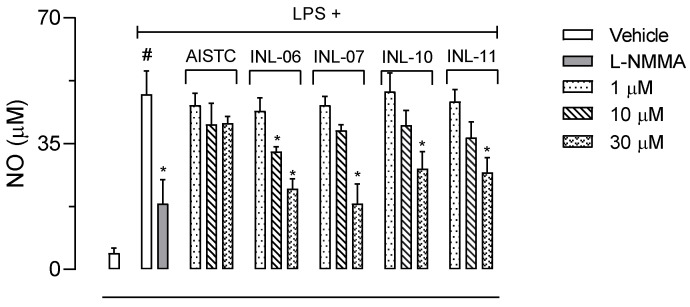
INLs inhibited nitric oxide (NO) production. Here, RAW 264.A1 cells were incubated with different concentrations of AISTC or INLs and, after 1 h, received lipopolysaccharide (LPS, 1 µg/mL). After 24 h, the supernatants were collected for NO measurement. Results are expressed as media ± standard error (*n =* 5). Statistical analyses were carried out using ANOVA following Newman–Keuls as the post-test. Here, # *p* < 0.001 when comparing with the vehicle-treated group, and * *p* < 0.01 when comparing the AISTC- or INLs-treated groups + LPS with LPS-treated groups.

The next step was to incubate cells with INLs at different concentrations and activated with LPS (1 µg/mL). After 24 h, cell supernatants were collected to several dosages. Figure 4 shows that non-activated cells produce 4.6 ± 1.4 µM of NO. After activation with LPS, cells increased their NO production 10.58-fold, reaching 48.7 ± 6.5 µM of the mediator. The inhibitor of the inducible nitric oxide synthase, L-NMMA, reduced NO production in 62% (18.3 ± 6.7 µM). Although AISTC did not affect NO production by cells, all INLs did significantly reduce the levels of NO. While INL-07, INL-10, and INL-11 inhibited only at 30 µM concentration, INL-06 presented a significant effect with the two higher concentrations used (10 and 30 µM).

The data obtained so far are suggestive that AISTC and INLs can inhibit NO production; however, we cannot conclude if this effect is due to inhibition in inducible nitric oxide synthase (iNOS) enzyme expression, its activity, or a direct NO-scavenger effect of each substance. 

**Figure 5:**
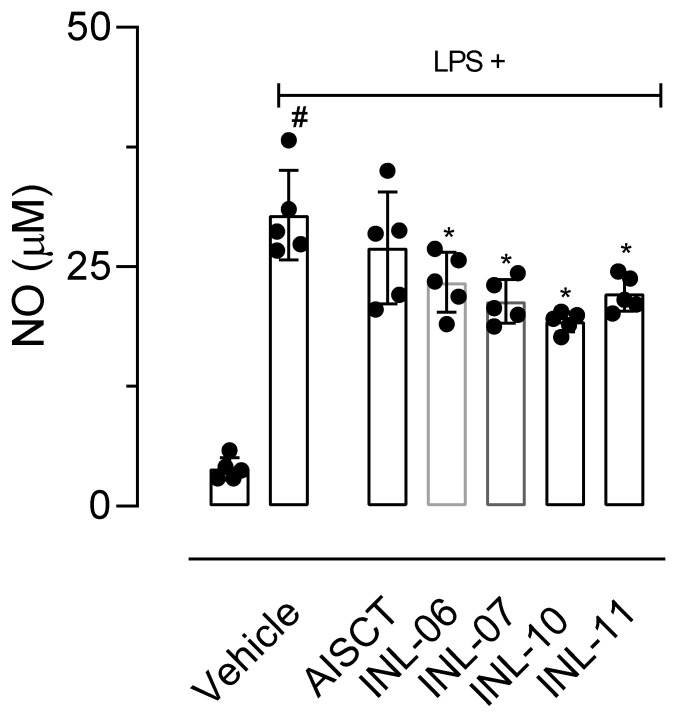
Effect of AISTC and INLs in inducible nitric oxide synthase enzyme activity. Here, RAW 264.7 cells were incubated with vehicle or lipopolysaccharide (LPS, 1 µg/mL) and, after 8 h, received AISTC or INLs (10 µM). Twenty-four hours after LPS activation NO accumulated in supernatant was measured. Results are expressed as media ± standard error (*n =* 5–7). Statistical significance was calculated in GraphPad Prism 8.0 (San Diego, CA, USA) using analyses of variance (ANOVA) followed by Tukey’s post-hoc test with *p* < 0.01 (#) when comparing LPS-activated cells treated with vehicle and non-activated cells treated with vehicle. Here, (*) refers to a comparison between LPS-activated cells treated with AISTC or INLs and LPS-activated cells treated with vehicle.

Trying to elucidate these possibilities, we first incubated LPS-activated cells with the substances and, after 8 h of activation, a period where protein synthesis of iNOS was finished and the enzyme begins it activity, each substance was added. After 24 h of activation, the supernatants were collected, and NO was measured. Results shown in Figure 5 demonstrated that INLs affected the NO production when added 8 h post-LPS activation, suggesting that these substances can influence enzyme activity.

**Figure 6:**
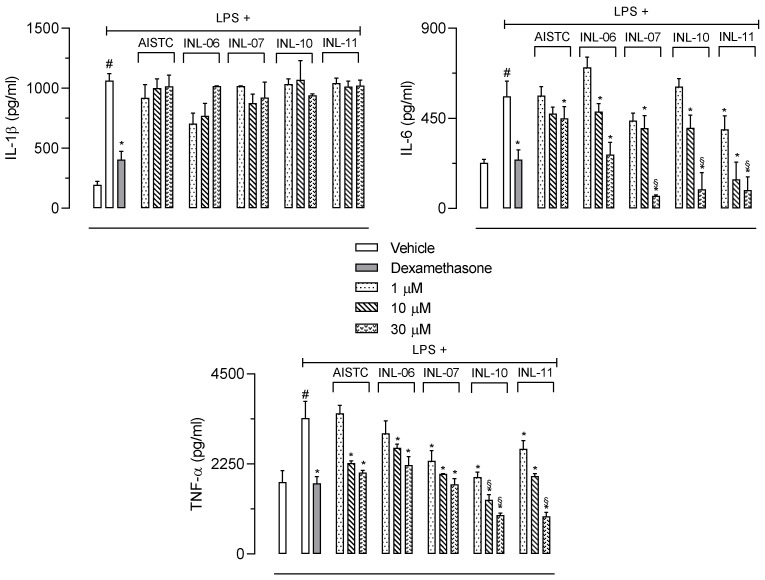
INLs inhibited cytokines production in vitro Here, RAW 264.7 cells were incubated with different concentrations of AISTC or INLs and, after 1 h, received lipopolysaccharide (1 µg/mL). After 24 h, the supernatants were collected for IL-1β, IL-6, or TNF-α measurement. Results are expressed as media ± standard error (*n =* 5). Statistical analyses were obtained using ANOVA following Newman–Keuls as a post-test. Here, # *p* < 0.001 when comparing with the vehicle-treated group, and * *p* < 0.01 when comparing AISTC- or INLs-treated groups + LPS with the LPS-treated groups. ^§^ *p* < 0.01 when comparing AISTC- or INLs-treated groups + LPS with the LPS-treated group with dexamethasone.

We also measured cytokines produced by RAW 264.7 cells after activation with LPS. Our data demonstrated that LPS activation resulted in high levels of IL-1β, IL-6, and TNF-α production after 24 h (1065 ± 55.8; 559.3 ± 76.7; 3399 ± 414.1 pg/mL, respectively). When cells were preincubated with different concentrations of each INL it could be noted that none of the substances affected IL-1β production. On the other hand, all INLs significantly reduced IL-6 production. The higher concentrations of INL-07, INL-10, and INL-11 drastically reduced the levels of IL-6 even when compared with the positive control group (activated cells preincubated with dexamethasone). The same three INLs also reduced the TNF-α production. It is worth mentioning that all three concentrations used (1, 10, and 30 µM) significantly inhibited the cytokines at the higher concentration (Figure 6).

### 3.6. INLs Reduced Cell Migration In Vitro

**Figure 7:**
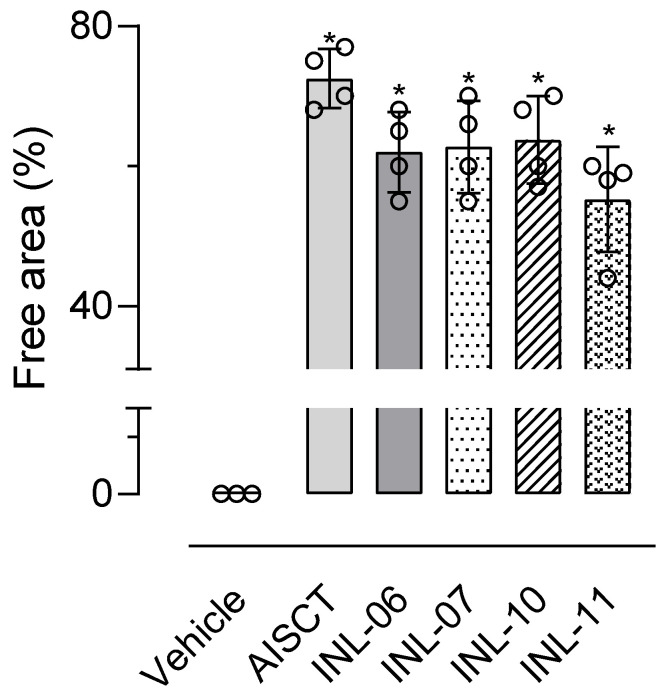
INLs inhibited RAW 264.7 cells migration in vitro. In a confluent cell monolayer, a healing was carried out with a tip. After 24 h, the healing capacity of cells was quantified by measuring the cell-free area using a microscope. Results are expressed as media ± standard error (*n =* 4). Statistical analyses were carried out using ANOVA following Newman–Keuls as a post-test. Here, * *p* < 0.01 when comparing each group at 24 h with their respective group at 0 h.

Since our data suggest that INLs reduce cell migration into the subcutaneous air pouch after injection of an inflammatory agent, we decided to test if the substances could present a similar effect in vitro. For this purpose, we used the wound healing model. In this model, data acquisition indicates the cell-free area after 24 h of incubation with INLs and LPS. Our results indicate that INL-11 was the substance that most inhibited cell migration. After 24 h in INL-11-treated cells, the percentage area remaining free of cells was 55% of the total. All other INLs also reduced/inhibited cell migration, and AISTC was the substance with the lowest ability to inhibit cell migration (Figure 7).

## 4. Discussion

Non-steroidal anti-inflammatory drugs are one of the most prescribed classes of drugs worldwide and are used to treat pain and acute inflammation; they are also used to treat chronic diseases, such as osteoarthritis, rheumatoid arthritis, and musculoskeletal disorders [19]. However, the drugs of this class currently available on the market cause many adverse effects that harm the patient’s life, leading to limitations in use. Thus, searching for new drugs with better therapeutic potential is a goal of several groups. Our data suggest, for the first time, the phenylhydrazide molecules INL-10 and INL-11 as anti-inflammatory substances with potential for further studies.

Our data demonstrated that AISTC as well as INLs reduced in a dose-dependent manner the migration of leukocytes into the SAP. In this model, at 24 h-post carrageenan injection, there are a high number of neutrophils accumulated in the exudate. Our results agree with the literature demonstrating that cellular reactions in inflammatory responses are seen within hours, with neutrophils being the first cells attracted to the site of injury [20,21,22]. Although neutrophils are the first line cells in the process, they also contribute to inflammation by releasing inflammatory mediators, including those that attract macrophages to the site of inflammation [21,23]. 

The inflammatory processes evoked by carrageenan also involve an increase in vascular permeability. Among diverse inflammatory mediators that can induce vascular permeability, it is well known that nitric oxide (NO) and PGE2 are major factors involved in the pathogenesis of many inflammation-associated diseases. The result is an increase in protein leakage and the accumulation of NO and cytokines in the exudate [24]. The INLs also drastically reduced the increase in vascular permeability, observed as an increase in protein leakage to the exudate. The effect observed was even greater than in the positive control group, dexamethasone. 

As TNF-NO and COX2-PGE2 pathways are considered the two main pathways of inflammatory processes, and they are blocked by inhibitors of iNOS (corticosteroids) and COX (NSAID), respectively [11,25], it could be expected that INLs could inhibit one of these mediators, thus, reducing the inflammatory phenomena. Several cytokines, particularly TNF-α and IL-1β, are known to play key roles in the induction and perpetuation of inflammation. Indeed, TNF-α is a cytokine that plays a key role in the innate immune response and is associated with reduced cell migration and exudation [26]. On the other hand, IL-1 β promotes the expression of adhesion molecules, leukocyte migration, and increased vascular permeability, indicating that it acts as an important pro-inflammatory mediator [27]. In this regard, we assess the effect of the substances under study in their ability to reduce the levels of those cytokines accumulated in the inflammatory exudates. When comparing all compounds tested, it could be noted that INL-10 and INL-11 were the most potent in inhibiting production of all cytokines. Moreover, INL-11 almost completely abolished TNF-α production. 

The inhibition of leukocyte migration observed in our study could also be related to a decrease in IL-1 β and TNF-α levels. These results are suggestive that the substances may be acting as modulators of the immune system by decreasing cell migration, exudation, and the production of immunomodulatory cytokines. Another possibility to explain the reduction in cytokine production could be a direct inhibition in leukocyte numbers migrating to the SAP. Thus, a parallel reduction in cell number could consequently reduce the number of cytokines produced. To exclude this hypothesis, we further used a cell culture of macrophages (RAW 264.7 cell line) to evaluate the direct effect of the substances against the cells. Similar to what was observed in vivo, INL-10 and INL-11 presented an inhibitory effect higher than the positive control group. In this system, NO inhibition was observed only with higher concentrations. These results corroborate those in vivo observations, suggesting that INLs reduce cell activity and not only cell motility and migratory capacity.

Salim et al. [26] described that after 8 h of activation with LPS there is a peak in the expression of the iNOS enzyme and that, despite this, the production of NO remains for 12 h. Thus, in a tentative effort to evaluate if INLs could present a direct effect on the enzyme, we evaluated the effect of INLs on NO production after 8 h of LPS activation. Data obtained showed that INLs affected NO production. These results suggest that the action of inhibiting the production of NO caused by INLs can occur through the reduction in the activity of this enzyme.

After obtaining positive results in reducing the production of inflammatory mediators in vitro, we chose to evaluate whether incubation with AISTC and INLs would influence the process of cell migration. Our assay is advantageous because it mimics the process of cell migration in vivo, in addition to being considered a simple and inexpensive technique to analyze this process [28]. The INL-10 and INL-11 inhibited at least 50% the migratory capacity of macrophages. This result can be explained, at least in part, by the role of INLs in the inhibition of NO.

According to Gao et al. [29], macrophages activated with LPS initiate the synthesis of a series of mediators, including the iNOS enzyme. The NO produced actively participates in the SRC-FAK cascade (steroid receptor co-activator–focal adhesion kinase). Studies indicate that this cascade is linked to the process of macrophage mobility, influencing their migratory capacity [29,30]. By understanding the importance of the SRC-FAK cascade in the migration process and how this cascade is highly dependent on NO, our hypothesis is that the effect caused by INLs to reduce cell migration could be due to the reduction caused in the production of NO.

It was observed that when the substituents were slightly electron-withdrawing halogens, such as bromine and chlorine (INL-07 and INL-09, respectively), the anti-inflammatory activity showed an increase. The introduction of methyl group (INL-11), an electron-donating group, increased activity. We described herein an improvement compared to the literature, in which there was no need for long reaction times [3,7,15,31]. Reactions were carried out catalyst-free [3,6], without the use of non-volatile solvents, such as DMF [6,32], and using an equimolar amount of substrate and nucleophile [3,7], thus, improving the synthesis of these molecules. 

## 5. Conclusions

Taken together our data suggest that the phenylbenzohydrazides synthetized present a significant anti-inflammatory effect in vivo as well as in vitro indicating that these compounds could be useful hits in the search for new analogues of new anti-inflammatory drugs.

## Data Availability

All data can be obtained directly from the authors.

## Sample Availability

Samples of the compounds (AISTC, INL-06, INL-07, INL-10, and INL-11) are available from the authors.
